# Evaluation of Enterprise Green Mine Construction Based on DPSIR Model

**DOI:** 10.3390/ijerph20064932

**Published:** 2023-03-10

**Authors:** Wei Yang, Mengge Xue, Yaping Wang, Tao Long, Sha Deng, Bo Deng, Nan Fang

**Affiliations:** 1 School of Resources Engineering, Xi’an University of Architecture and Technology, Xi’an 710055, China; 2 Key Laboratory of Gold and Resources in Shaanxi Province, Xi’an 710055, China; 3 Technology & Equipment Institute of Green Beneficiation-Metallurgy, Xi’an University of Architecture and Technology, Xi’an 710055, China; 4 School of Foreign Languages, Baoji University of Arts and Sciences, Baoji 721013, China

**Keywords:** DPSIR framework model, combination weighting, TOPSIS coupling coordination evaluation, main obstacle factors, sustainable development

## Abstract

As a new mode of mining development, green mine optimizes the development and utilization of mineral resources with a minimum of the environmental impact, and how to objectively evaluate the construction level of the green mine has become the key to promote green mine construction and it has also been an important path to achieve sustainable development of mineral resources. The evaluation system and methods of green mine construction, however, are not perfect at present as the existing green mine evaluation mostly adopts the index scoring accumulation method, with which the internal relations between the indicators are ignored, and the subjective influence it causes is too large. Based on the framework model of driving forces, pressure, state, impact and response, an indicator system is constructed in this paper to express the internal relationship between indicators more intuitively. Combined with subjective and objective combination weighting method to determine the index weight, TOPSIS and coupling coordination degree models are introduced to quantitatively evaluate the spatio-temporal evolution process of green mine construction and the coupling coordination between subsystems, analyze and obtain the main obstacle factors affecting the green mine construction of enterprises, and provide suggestions and countermeasures for the improvement of green mine construction of enterprises. The applicability of the model is verified by an actual case study of a mine in China. The model enriches the connotation of green mines, making the evaluation process and results fairer and more reliable, thus providing an effective way to promote the sustainable development of mines.

## 1. Introduction

It is a necessary way to realize the rational and efficient utilization of natural resources and the healthy and orderly progress of the sustainable development and production of mineral resources, and the construction of “*green mines*” can accelerate the sustainable development of the mining industry [1,2]. The expression green mines has more meanings than just “*greenified mines*”, which has such further meanings as mining, production, management, environmental protection, resource utilization, scientific and technological innovation, community harmony, and corporate culture, etc. It can be said that green mine construction runs through the whole process of mine development, such as mine planning, design, construction, operation, and pit closure etc., so it is of great significance to balance resource and ecological environment protection [3,4].

As a complex system, the green mine and its construction and improvement are still in the exploration stage [5,6]. A standard system needs to be established to refine the factors in the system, so as to provide guidance and countermeasures for enterprises [7]. Huang Jingjun [8] believed that the construction of green mines should emphasize resource conservation, environmental friendliness, the respect for the original natural ecology, saving of the mineral resources, and protection and reconstruction of the landscape ecology. Lai Xiaoying [9] constructed a green mine index system from the aspects of efficient utilization of resources, energy conservation and emission reduction, standardized management, production safety and environmental reconstruction. With the continuous deepening of the research, some scholars [10] believed that in the period of emphasizing environmental friendliness, special attention should also be paid to economic development, so as to maintain a certain degree of coordination between the ecologic and the economic systems formed by mine development. Zhang Yingliang [11] proposed, from the life cycle of mine construction, that the whole life cycle of mine should be coordinated with the surrounding environment, and meet the requirements of sustainable development. Some scholars, with the introduction of environmental costs into mining activities, implemented the green sustainable development strategy by analyzing the environmental problems arising from mining, concentration and mineral processing operations [12]. From different perspectives, a multi-objective and multi-level evaluation index system was established in these studies, and the influencing factors related to the green mine construction of enterprises were determined. However, the internal links between these factors were ignored, which caused incomplete description of the structure and function of each component.

The evaluation of the construction degree of enterprises’ green mines is helpful in understanding the construction situation of enterprises more intuitively and efficiently, and it can provide guiding countermeasures for the comprehensive construction of green mines [13]. At present, the indicator scoring accumulation method is often adopted in practice to evaluate the construction of enterprises’ green mines. That is, by accumulating the scores of experts, it can be determined whether the indicators meet the national green mine construction standards [14]. The method, despite the advantages of simple operation, has such obvious problems as a large impact of subjective factors and the lack of objectivity in evaluation process [15]. With the deepening of research, such methods as analytic hierarchy process, principal component analysis, fuzzy comprehensive evaluation, data envelopment analysis, etc. were introduced into the green mine evaluation process to improve the science and precision of the evaluation system [16,17,18]. Song Ziling [19] and other scholars put forward the concept of “green degree”. With the concept and the evaluation criteria, the current green mining degree of mining enterprises was evaluated. Weizhang Liang [20] made a comparative evaluation of the sustainability of the four mines based on the mixed multi-criteria method. These evaluations, with the external conditions as a reference, evaluated the construction situation at a certain time node. Yet due to the different scale, output and technical level of mining enterprises, the development focuses on different directions. Only by evaluating the enterprise’s own construction situation and development trend can suggestions and countermeasures be put forward with strong applicability.

In view of the above discussion, there are still some research gaps in the existing literature. On the one hand, in the process of establishing the indicator system, in most studies, the internal relations between indicators were ignored, and only the influencing factors related to green mine construction in general were listed. On the other hand, most of the established evaluation models only evaluate and analyze the construction effect at a certain time point, but fail to focus on external comparison and ignore the change and development trend between years of the enterprise and the evaluation of the internal coordination of the system. The evaluation of the enterprise’s green mine construction is not comprehensive, and it is difficult to provide guidance for future construction and development.

With the increasing perfection of the research on the evaluation system and model of green mine construction, there still exist some deficiencies. In order to supplement the deficiencies of existing research, the DPSIR framework is applied in this paper to build an indicator system for the illustration of the mechanism of each element. In building the evaluation model, the subjective and objective combination weighting method is introduced so as to make the index weight meet the subjective expectations of the decision-makers and the objective judgment requirements of the inconsistency between the indicators, and based on time series, the driving forces, pressure, state, impact, response to each subsystem and the internal coordination of the system are comprehensively evaluated so that the main obstacles to the enterprise’s green mine construction are identified through evaluation and analysis. Finally, the applicability of the model is verified by an example mine for the scientificity of the evaluation of green mines. The purpose of this research is to improve the evaluation criteria of green mines, optimize the evaluation methods, and provide decision-making basis for the green development strategy of mining enterprises.

## 2. Research Design

### 2.1. Design Principles of the Evaluation Index System

In the design process, the principle of sustainable development is chiefly applied in the evaluation index system with the emphasis of the combination of efficient utilization of resources, safety and environmental protection in the production process and optimal management of the mining area environment [21,22]. With the implementation of waste reduction, harmless treatment, and rational and efficient utilization of resources in the whole process of mine production, green mining and clean production is promoted [23,24]. Therefore, an index system suitable for internal evaluation of enterprise green mines can be established from the aspects of mining area environment, resource development mode, comprehensive utilization of resources, energy conservation and emission reduction, scientific and technological innovation and intelligent mines, enterprise management and enterprise image etc.

### 2.2. Evaluation System of Green Mine Construction Based on DPSIR Model

The DPSIR model (Driving forces-Pressure-State-Impact-Response) is proposed by the European Environment Agency (EEA) on the basis of PSR (Pressure-State-Response) and DSR (Driving forces-State-Response) models. As a causal framework, the DPSIR model systematically summarizes the interaction and relationship between human and natural environment with the characteristics of integrity, continuity, and operability. This model reflects not only the impact of social and economic development and human behavior on the ecological environment, but also the feedback of human behavior and the resulting environmental state on society [25]. The model is widely used in quality management and sustainable assessment in the fields of environmental protection, urban integrated management and resource carrying capacity, etc. [26,27,28]. Green mining is a complex system with comprehensive consideration of ecological, social, and economic benefits and the DPSIR model provides a broad analysis framework and can effectively integrate information. Therefore, the combination of the system and the model will help to map the causal relationship of the green mining system more accurately.

**Figure 1:**
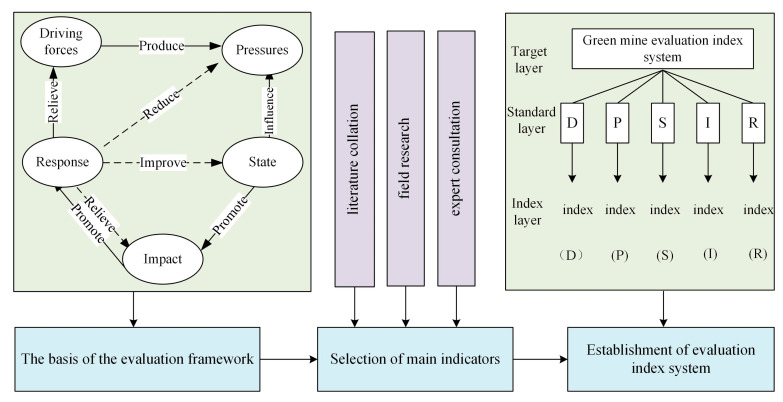
Green Mine Construction Evaluation System Based on DPSIR Framework.

Such important elements are included in the DPSIR model as socio-economic growth, environmental pollution, ecological destruction, resources and environmental conditions, the impact on the environment and society, and solutions to improve efficiency. The Index System based on DPSIR framework can essentially show the interaction between mineral resources development and ecological environment in the process of enterprise economic development, ecological environment damages, the changes of environmental quality in mining areas, objective reflection of the green mine construction and effect on the actual impact of enterprises. Based on the driving forces, pressure, state, impact and response, a green mine construction evaluation index system is founded in this paper together with the combination of qualitative and quantitative indexes. (see Figure 1).

## 3. Materials and Methods

**Figure 2:**
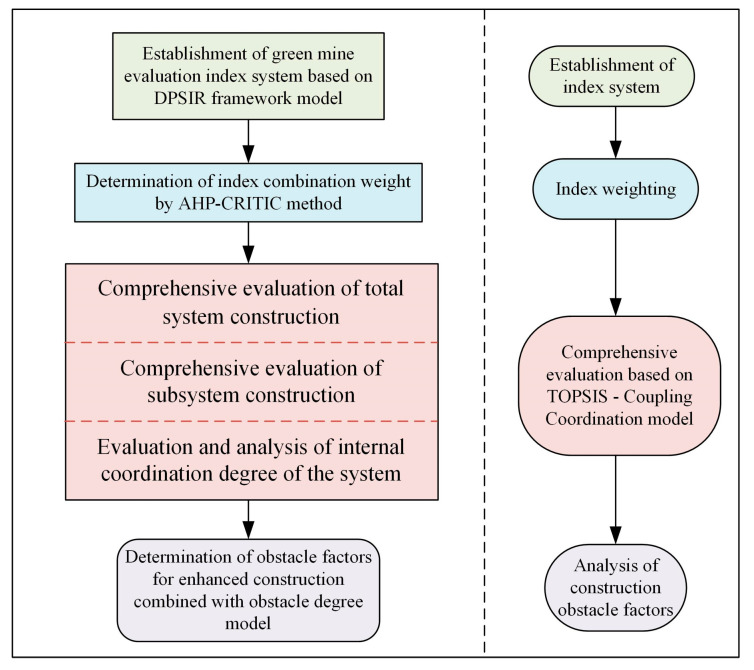
Flow Chart of Green Mine Assessment.

The index system is established on the DPSIR framework model, and the index is weighted by combining the subjective and objective combination weighting method. In evaluating the effectiveness of mine construction, the subsystems of the driving forces, pressure, state, impact, response and the overall green mine system are evaluated respectively with time as a sequence and the combination of TOPSIS evaluation method. At the same time, the internal coordination of the system is evaluated for the main obstacle factors affecting the enterprise construction. The evaluation process is shown in Figure 2.

### 3.1. Index Decomposition Based on DPSIR Framework

In the DPSIR conceptual model, such factors as resources, society, economy, environment, technology and others are integrated into the indicator system, forming a close causal chain to clarify the interaction within the green mine system. The driving forces are adopted for the description of the potential power to promote the construction of green mines, in which the factors are included that promote enterprises to improve economic benefits. Pressure, as not only a specific human activity, but also a natural process, is mainly reflected in the production capacity of the mine and energy consumption in the production process and in the mine production activities on the ecological environment. The state is the current resource and environment situation of green mine construction. The impact, as the result of the interaction of the above three parameters, refers to the impact of green mine construction on the enterprise’s economy and image. Response, related to decision-making, aims to control driving factors to maintain status or gain improvement, and help adapt to the impact. On the basis of the principles of systematicness and independence, a green mine evaluation index system consisting of 5 first level indicators and 20 s level indicators is established.

**Figure 3:**
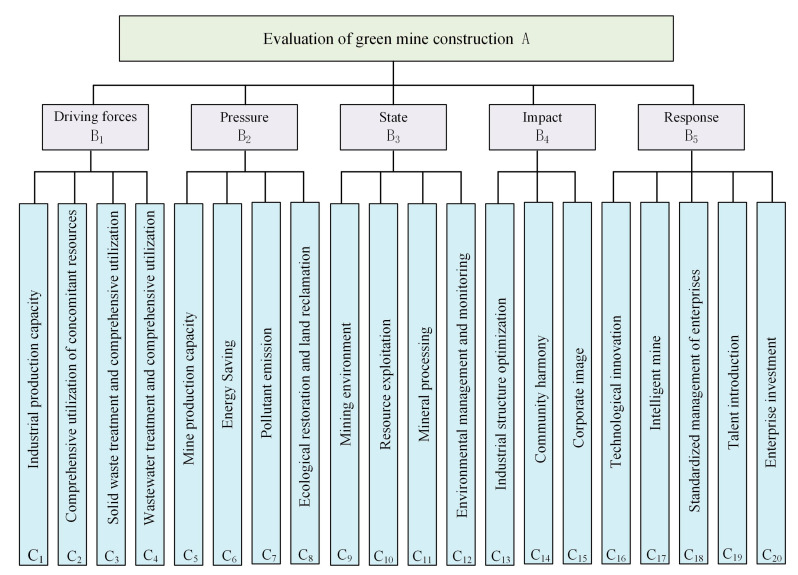
Green mine evaluation index system.

Therefore, the evaluation index system of green mine construction is established in Figure 3.

### 3.2. Calculation of Combination Weighting

After the establishment of the green mine evaluation index system, it is necessary to quantify the weight of each index. In this paper, AHP and CRITIC methods are applied in the subjective and objective combination weighting. The evaluation of green mines is a huge systematic project. The weight obtained from subjective factors alone is not operable, and the uncertainty in the expert evaluation process and the difference of index scores are not taken into consideration in the weight distribution. The evaluation results are one-sided and subjective. However, in the specific practice process, the CRITIC method can be used to obtain more objective index weights. With the combination of the two evaluation methods, reasonable weight of indicators can be achieved.

#### 3.2.1. Determination of Subjective Weight by Analytic Hierarchy Process (AHP)

Analytic Hierarchy Process (AHP) is a multi-criteria decision analysis method proposed by Saaty in 1970 [29]. In the multi-objective decision making, a qualitative way is mainly applied in evaluation with the analytic hierarchy process (AHP). In this method, the multi factor decision-making is transformed into a multi-level single factor problem to hierarchize the complex decision-making system, and to replace human subjective judgment with data expression [30].

The specific evaluation steps are as follows:

(1) Establishment of the initial judgment matrix

In the analysis of the target problem, if there are *m* evaluation objectives and each evaluation target has *n* evaluation indicators, the judgment matrix will be constructed as follows:

(1) $$D=\left[\begin{array}{ccc} \begin{array}{ccc} x_{11} & \cdots & x_{1j} \end{array} & \cdots & x_{1n}\\ \begin{array}{ccc} \vdots & \cdots & \vdots \end{array} & \cdots & \vdots \\ \begin{array}{ccc} \begin{array}{c} x_{i1}\\ \vdots \\ x_{m1} \end{array} & \begin{array}{c} \cdots \\ \cdots \\ \cdots \end{array} & \begin{array}{c} x_{ij}\\ \vdots \\ x_{mj} \end{array} \end{array} & \begin{array}{c} \cdots \\ \cdots \\ \cdots \end{array} & \begin{array}{c} x_{in}\\ \vdots \\ x_{mn} \end{array} \end{array}\right]=\left[\begin{array}{c} \begin{array}{c} D_{1}\left(X_{1}\right)\\ \vdots \\ D_{i}\left(X_{i}\right) \end{array}\\ \vdots \\ D_{m}\left(X_{m}\right) \end{array}\right]$$

where *D* is the evaluation target; $X_{1},\;\;X_{i}$ and $X_{m}$ are the evaluation scheme; $\;x_{11}$, $x_{ij\;}$ and $x_{mn}$ are the evaluation index; $x_{ij}$ represents the degree of importance of factor *i* compared to factor *j*, which is usually quantified from 1 to 9 scale in Analytic Hierarchy Process (see Table 1).

(2) Normalization of judgment matrix

First, the data in the index system is processed by standardization processing for the indicators.

(2) $${\bar{x}}_{ij}=\frac{x_{ij}}{\sum_{k=1}^{n}x_{kj}}\;(i=1,2,\ldots ,m;\;j=1,2,\ldots ,\;n)$$

Then the weight coefficient is achieved with the standard judgment matrix.

(3) $$u_{i}=\frac{\bar{W_{i}}}{\sum_{i=1}^{m}\bar{W_{i}}}\;$$

where

(4) $$\bar{W_{i}}=\sum_{k=1}^{n}{\bar{x}}_{ij}$$

and $W_{i}\;$ is the index weight.

(3) Calculation of the eigenvalue

(5) $$\lambda_{\max}=\frac{1}{n}\overset{n}{\underset{i=1}{\sum }}\frac{{\left(DW\right)}_{i}}{W_{i}}$$

where $\lambda_{\max}$ is the maximum eigenvalue of the judgment matrix.

(4) Consistency test

(6) $$CI=\frac{\lambda_{\max}-n}{n-1}$$

where n  represents the number of indicators. The corresponding *RI* values are determined according to the judgment matrix order, as is shown in Table 2.

Finally, the consistency proportion *CR* was calculated:

(7) $$CR=\frac{CI}{RI}$$

when *CR* < 0.1, the judgment matrix passed the consistency test; otherwise, it shows that it did not pass the test and the above procedure must be repeated until the requirement to meet the consistency test is achieved.

#### 3.2.2. The Determination of the Objective Weight with the CRITIC Method

The CRITIC method, (Criteria Importance Through Inter-criteria Correlation method) as an objective weight empowerment method, considers not only the influence of the index variation degree on the weight, but also the conflict between the indicators [31]. The degree of difference is expressed in the form of the standard deviation: the larger the standard deviation is, the larger the value difference is. The correlation is shown as the correlation coefficient, which has less conflict between the two features if there is a strong positive correlation. The method, combining the standard deviation and correlation coefficient, determines the amount of information contained in each attribute and thus achieves its weight [32].

The collected data should be normalized before the weight analysis. The smaller the index value of cost index, the better; while the larger the index value of benefit index, the better. The standardization of the two types of indicators are as follows:

Cost-type:

(8) $$\;b_{ij}=\frac{\max x_{i}-x_{ij}}{\max x_{i}-\min x_{i}}$$

Benefit-type:

(9) $$\;b_{ij}=\frac{x_{ij}-\min x_{i}}{\max x_{i}-\min x_{i}}$$

(10) $$\overset{n}{\underset{i=1}{\sum }}\left(1-r_{ij}\right)$$

In the CRITIC weight analysis method, the Equation (10) is applied as a standard to measure the contradiction of the two indicators, and to indicate the amount of information contained in the first indicator $\;I_{i}$.

(11) $$I_{i}=\rho_{i}\sum_{i=1}^{n}\left(1-r_{ij}\right)$$

Here,

(12) $$\rho_{i}=\frac{\sigma_{i}}{{\bar{X}}_{i}}$$

The correlation coefficient is:

(13) $$r_{xy}=\frac{{\left(x-\bar{x}\right)}^{2}{\left(y-\bar{y}\right)}^{2}}{\sqrt{\sum^{\;}{\left(x-\bar{x}\right)}^{2}\sum^{\;}{\left(y-\bar{y}\right)}^{2}}}$$

In the Equation (13): $\sigma_{i}$ is the standard deviation; $\;{\bar{X}}_{i}$ is the average; $r_{ij}$ is the correlation coefficient; *x* and *y* are two sets of data; $\bar{x}$ and $\bar{y}$ and are the average of the two sets of data. Therefore, the bigger the number $I_{i}$ is, the more information this index contains, and thus the higher the importance in the actual multi-objective decision model is shown and the greater the weight coefficient should be given. The index weight is calculated as follows:

(14) $$v_{i}=\frac{I_{i}}{\sum_{i=1}^{n}I_{i}}$$

#### 3.2.3. Determination of Comprehensive Weight by AHP-CRITIC Method

The correlation is analyzed between the subjective weight calculated by AHP method and the objective weight by CRITIC method. If there is no relation (*p* > 0.05), it means that the contents reflected by the two methods are independent and unrepeatable.

As a method of subjective randomness, AHP method cannot fully reflect the scientific requirements, while as an objective method, CRITIC evaluation cannot reflect the experience and opinions despite a strong objectivity [33]. Therefore, in order to show the influence of the subjective and objective weights, the subjective weight $u_{i}$ obtained by AHP and the objective weight $v_{i}$ by CRITIC method are applied to achieve the comprehensive weight $\omega_{i}$. (See Equation (15)).

(15) $$\omega_{i}=au_{i}+\left(1-a\right)v_{i},\;\;\;\left(0\leq a\leq 1\right)$$

With the consideration of the proportion of subjective and objective indicators in the evaluation practice of green mine construction, *a* value is set at 0.7, that is, the proportion of subjective weight used by AHP method is 0.7, and that by CRITIC method is set at 0.3.

### 3.3. Comprehensive Evaluation and Analysis

With the time series as the benchmark, first, the degree of green mine construction of the overall system and subsystems is evaluated through TOPSIS model. Then, the degree of coordination between the subsystems is evaluated with the coupling coordination model. Finally, through the obstacle degree function, the main obstacle factors in the enterprise’s production process are determined for the guidance of the enterprise to promote green mine construction.

#### 3.3.1. TOPSIS Model

TOPSIS (Technique for Order Preference by Similarity to an Ideal Solution), also known as the “ranking method of approaching ideal solutions”, was first proposed by C. L. Hwang and K Yoon in 1981, which mainly uses the distance principle to solve the common multi-objective decision-making analysis problems in life. That is, with the calculation of the European distance between different evaluation units and positive ideal solutions and negative ideal solutions, if the closer the result is to 1, the better the evaluation unit will be [34].

The weight normalization matrix *S* is calculated from the data obtained by normalization and the composite weight by combination weighting:

(16) $$S={\left(s_{ij}\right)}_{m\times n}$$

(17) $$s_{ij}=\omega_{i}\times d_{ij}$$

Next, the positive ideal solution $S_{j}^{+}$ and negative ideal solution $S_{j}^{-}$ of each index are determined:

(18) $$S_{j}^{+}=\max\left\{S_{1j},\;S_{2j},\;S_{3j},\;\cdots ,\;S_{ij}\right\}$$

(19) $$S_{j}^{-}=\min\left\{S_{1j},\;S_{2j},\;S_{3j},\;\cdots ,\;S_{ij}\right\}$$

Then, the sums of Euclidean distance $d_{j}^{+}$ and $d_{j}^{-}$ are calculated between each evaluation unit and positive and negative ideal solutions:

Distance of Positive ideal solution:

(20) $$d_{j}^{+}=\sqrt{\sum_{j=1}^{n}{\left(s_{ij}-S_{j}^{+}\right)}^{2}}$$

Distance of negative ideal solution:

(21) $$d_{j}^{-}=\sqrt{\sum_{j=1}^{n}{\left(s_{ij}-S_{j}^{-}\right)}^{2}}$$

Finally, the closeness is calculated:

(22) $$C_{i}=d_{j}^{-}/\left(d_{j}^{-}+d_{j}^{+}\right)\;\;\left(i=1,\;2,\;3,\ldots ,m\right)$$

where $C_{i}$ represents the degree of green mine construction of the enterprise in the *i* year: 0 ≤ $\;C_{i}$ ≤ 1, meaning that the greater the $C_{i}$ is, the better the degree of green mine construction will be. With this same method, the closeness of the five subsystems can be achieved.

#### 3.3.2. Calculate the Coupling Co-Scheduling of All Subsystems

As a composite system with multiple subsystems coupling the green mine system is, in which the coupling coordination degree model in physics is introduced, so that the degree of interaction and interaction among the five subsystems of driving forces, pressure, state, impact and response can be quantitatively analyzed and calculated (Table 3). The specific calculation formula is as follows:

(23) $$U=5\times {\left(\frac{C_{1}\times C_{2}\times C_{3}\times C_{4}\times C_{5}}{\prod^{\;}\left(C_{1}+C_{2}+C_{3}+C_{4}+C_{5}\right)}\right)}^{\frac{1}{5}}$$

(24) $$D=\sqrt{U\times T}$$

(25) $$T=aC_{1}+bC_{2}+cC_{3}+dC_{4}+eC_{5}$$

where, $\;C_{1},\;\;C_{2},\;\;C_{3}$, $C_{4}$, and $C_{5}$ respectively represent the closeness of driving forces, pressure, state, impact and response subsystems; *U* is the coupling degree; *T* is the degree of development; *a*, *b*, *c*, *d*, and *e* represent the weight coefficients of the five criteria layers respectively; and *D* refers to coupling coordination value.

#### 3.3.3. Obstacle Function Model

As a mathematical model for obstacle diagnosis based on the deviation degree of indicators, the obstacle function is applied to determine the obstacle degree of each indicator by calculating the deviation degree between the indicator and the optimal value. Its advantages are that it is not affected by subjective factors, and its calculation amount is small, while its disadvantages are that it cannot reflect the dynamic changes of the targets [35]. The introduction of the obstacle function model on the research is to identify the main obstacle factors affecting the green mine construction of enterprises:

(26) $$P_{ij}=\frac{\omega_{j}\times \left(1-d_{ij}\right)}{\sum_{j=1}^{20}\omega_{j}\times \left(1-d_{ij}\right)}$$

where:$\;P_{ij}$ represents the obstacle degree of the *j*th index in the *i*th year, and $\omega_{j}$ represents the weight of the index.

## 4. Case Study

### 4.1. Overview of the Gold Mine

The case mine, located in Fengxian County Shaanxi province with a mining range of 1310 m–1020 m and mining area of 0.1273 km^2^, is a gold mine with a production scale of 6.010^4^ t/a. There are clear and reasonable overall layouts for the mining area, concentrator, tailings pond, office and living areas as well as other functional areas with complete facilities and good operation conditions.

### 4.2. Evaluation of the Green Mine Construction of a Gold Mine

#### 4.2.1. Index Weight Calculation

Through the preliminary investigation, the relevant quantitative indicator data of a gold mine in Shaanxi from 2017 to 2021 were collected, and then the qualitative indicators were evaluated and scored through questionnaires combined with the construction standards. Table 4 shows the results of the five-year data after data normalization.

Combined with Formulas (1)–(14), the subjective weight is calculated by AHP method, the objective weight is calculated by CRITIC method, and then combined with Formula (15), the comprehensive weight is obtained. The calculation results are shown in Table 5.

**Figure 4:**
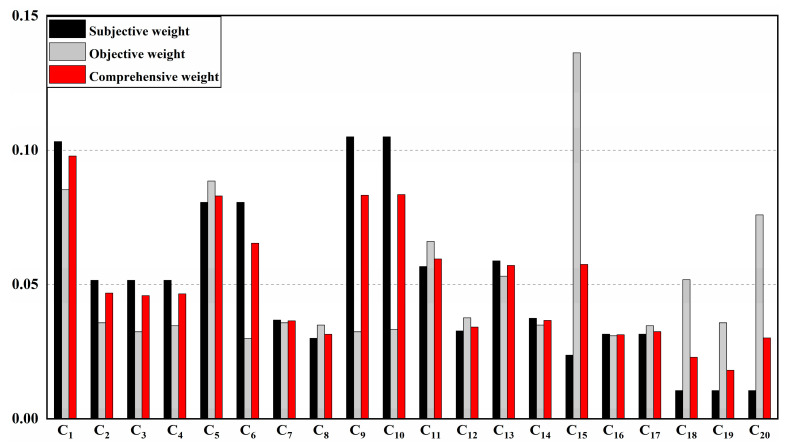
Comparison Chart of Secondary Indicator Weights.

For the correlation analysis of the calculated subjective and objective weights, the Pearson correlation coefficient is −0.041 between the weights obtained by CRITIC and AHP methods, indicating that the two are very weakly related or unrelated, that is: the contents reflected in the two empowerment methods are independent of each other and unrepeatable, as is shown in Figure 4.

It can be seen from Table 5 that there occupies a large weight in the regions of the industrial production capacity, mining environment, resource exploitation and mineral processing factors in the indicator system, indicating that enterprises should pay more attention to production capacity and resource exploitation and utilization level in green mine construction. In the process of promoting the sustainable development of mining industry, the improvement of the three rate indicators of the mine, the recovery rate of ore resources and the energy utilization rate should be emphasized and the dilution rate be reduced, so that the indicators can be continuously improved and the loss and dilution of the mine reduced. With the increasing demand for resources and the increasing pressure on the resource environment, resource-based enterprises must optimize their industrial structure according to their own actual conditions to achieve the transformation between solid wastes and raw materials, wastewater and production and domestic water, so that a closed-loop cycle of resources can be formed, thus enhancing the influence of the enterprise. The weight calculation results are consistent with the actual development of the enterprise, which verifies the feasibility of the model, and provides ideas for determining the index weight in the evaluation of green mine construction.

#### 4.2.2. Comprehensive Evaluation and Analysis

First, the weighted normalization matrix is obtained by using Formulas (16) and (17). Then, the distance between positive and negative ideal solutions is calculated by combining Formulas (18) and (21). Finally, the relative closeness is calculated by Formula (22) to evaluate the construction of green mines in enterprises. The calculation results are shown in Table 6.

It can be seen from the Table 6 that during the research period, the evaluation results of the enterprise’s green mine construction increased from 0.171 in 2017 to 0.770 in 2021, showing a trend of steady growth, which was mainly due to the strengthening of the enterprise’s green development concept, and the cleaner production concept as a guiding thinking guides the enterprise’s work. From 2019 to 2020, the evaluation results increased significantly from 0.416 to 0.615, and during this period, a lot of rectification was carried out in the mining enterprise, introducing advanced new technologies, new methods, and new processes of green exploration, improving the production efficiency with innovative ideas and methods, and greatly accelerating the construction of the enterprise’s green mine.

**Figure 5:**
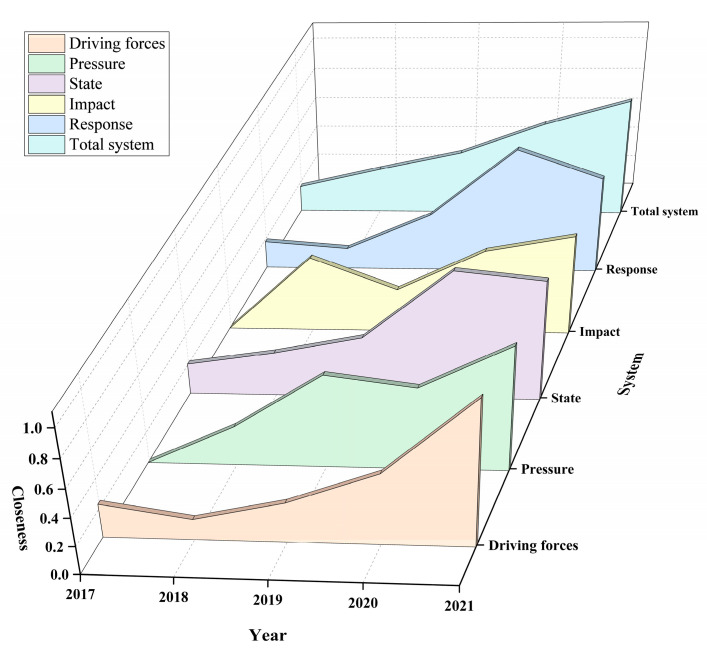
Change Trend of Closeness in 2017–2021.

Similarly, the change trend of closeness of each subsystem can be obtained as shown in the Figure 5.

**Figure 6:**
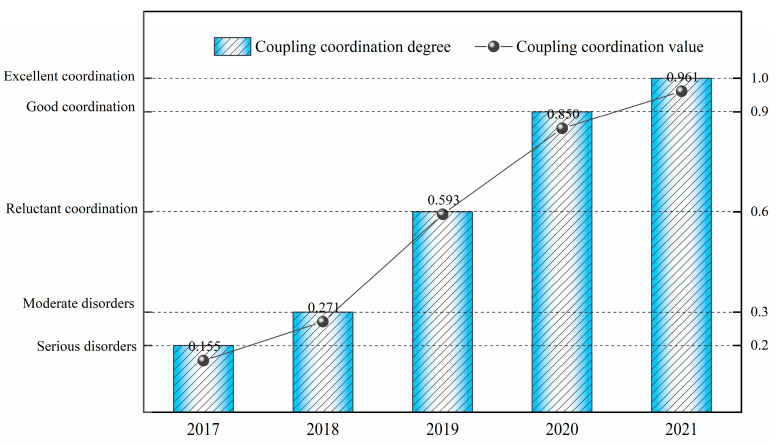
Change Trend of Coupling Co-scheduling in 2017–2021.

It can be seen in Figure 5 that the closeness of each subsystem fluctuated and increased in 2017–2021. The overall stable growth of the driving force subsystem is mainly due to the main indicators of the driving force subsystem: the production capacity and resource utilization in which the increasement has always been the focus of enterprise development. From 2019 to 2020, the construction of the status subsystem has been increased from 0.411 to 0.863, meaning that the technological upgrading and transformation of the enterprise has achieved remarkable results. At the same time, the pressure subsystem has been reduced from 0.634 to 0.559, indicating that while the scale of resource exploitation and mining has increased and consequently, the pressure on ecological restoration and energy saving and consumption reduction has also increased. The overall fluctuation of the impact subsystem is obvious, which, with close relation to community harmony and participation in public welfare undertakings, has a large subjective impact. There is a good agreement of the fluctuation range between the response and the state subsystem, and the response changes with the change of the state subsystem. Both the response and the status subsystem have a certain downward trend from 2020 to 2021, indicating that there is a certain slack after the completion of technical transformation. Therefore, the work of maintaining green and protecting green in the mine also needs to be taken into consideration as a focus. Then, the coupling coordination among the five subsystems of driving forces, pressure, state, impact and response is determined through Formulas (23)–(25). The calculation results are shown in Figure 6.

The green mine is a complex system, in which the subsystems interact with one another, so the coordinated development of the systems is very important. It can be seen from Figure 5 that from 2017 to 2021, with the continuous strengthening of technological innovation, the promotion of digital management, and the strengthening of the concept of ecological development, the coupling and coordination index among subsystems has steadily increased. The mining enterprise has transformed from the traditional development and utilization of mineral resources from consuming mineral resources and destroying the ecological environment to the development and utilization with quality and efficiency. This reform has successfully realized the green transformation of the enterprise and made it steadily develop towards the sustainable direction. Finally, the main obstacle factors restraining the development of enterprises are calculated through the obstacle function model. The calculation results are shown in the Table 7.

It can be seen from the Table 7 that the industrial production capacity ($C_{1}$), the mine production capacity ($C_{5}$), the mining environment ($C_{9}$) and the mineral processing ($C_{11}$) are the main obstacles in inhibiting the mining enterprises from building green mines. With the continuous construction and improvement of the enterprise, the idea of “production, construction and reclamation at the same time” has been realized, and the mine environment has been significantly improved. Through the renovation and transformation of energy-saving and consumption reducing equipment, the utilization rate of energy has been improved, providing a strong support for the growth of mining enterprises. Till 2021, these major obstacles obtained some improvement to some extent.

With the analysis of the construction obstacles of enterprises in 2021, it is found that such factors are in urgent need of improvement as industrial structure optimization ($C_{13}$), enterprise investment ($C_{20}$), mineral processing ($C_{11}$), mine production capacity ($C_{5}$), etc. In the subsequent construction and development of enterprises, the optimization of industrial structure can be taken as the focus of construction, in which the integration of mineral resources, the optimization of management mode, the resource scale structure and the reorganization of production factors can be completed so as to realize intensive operation and large-scale production. In the enterprise investment, research and development funds should be appropriately increased so as to introduce advanced technology, optimize process flow, and reduce mine loss and dilution. In terms of improving the level of mineral processing, low-grade minerals should be fully recycled to make comprehensive use of mineral resources so as to achieve the optimization of resource utilization. In order to improve the mine production capacity, enterprises should take into account the factors of investment, economy, equipment capacity, market risk, and conduct a comprehensive evaluation of the feasibility and economy of each plan, so as to realize the scientific decision of resource development.

## 5. Conclusions

Based on the DPSIR framework model, in this paper a qualitative and quantitative evaluation index system is constructed for green mine construction, and it accurately shows the chain feedback mechanism as among the indicators of a green mine. In the construction of the evaluation model, with the combination of weights, the defect is improved that subjectivity affects the evaluation results in the traditional evaluation; based on TOPSIS method, the coordination of the green mine system and its subsystems is evaluated, and the coordination between the established index systems is more intuitively reflected; analyzes the obstacle factors in the process of green mine construction is analyzed in the research and the improvement direction for the green mine construction of enterprises put forward, making the evaluation model more practical.

With the comprehensive evaluation of a gold mine in Shaanxi, China, it is concluded that the comprehensive evaluation value of green mine construction of the mining enterprise increased from 0.171 to 0.770 in 2017–2021, and the system coupling coordination degree increased from 0.155 to 0.961, showing a trend of steady growth. In addition, combining with the obstacle function model, the obstacle factors for the development of the enterprise are determined, providing phased suggestions for the future development of the enterprise. Through evaluation and analysis, the feasibility of the evaluation index system and evaluation method applied in this paper is verified with the combination of theoretical research and case study.

The following aspects of this study still need to be further improved and supplemented. First of all, in the selection of evaluation indicators, only secondary indicators are adopted and 20 indicators are selected, which limits the reliability of the results to a certain extent. In the future research, the indicators can be further refined in combination with the characteristics of mining enterprises to make the evaluation more scientific and comprehensive. Secondly, in the aspect of satisfactory consistency test of analytic hierarchy process, the commonly used threshold is selected in this paper, and the views of other scholars are not analyzed or discussed. In the future research, it is necessary to establish satisfactory consistency test standards that adapt to the characteristics of mining enterprises so that the scientific accuracy of green mine evaluation can be improved.

Due to the limitation of natural environment and resource conditions and different characteristics of mining enterprises, further researches should be conducted on how to build a model to combine the internal evaluation and external comparison of mining enterprises more closely. The model, in improving the evaluation of green mine construction and providing countermeasures and suggestions for enterprises, is expected to offer a direction for future efforts on promoting the sustainable development of mining industry.

## Figures and Tables

**Table 1:** Index comparison importance level table.

Definition	Intensity of Importance
Equally important	1
Moderately important	3
Strongly more important	5
Very strongly more important	7
Extremely more important	9
Intermediate values between the two adjacent judgments	2, 4, 6, 8

**Table 2:** Values of the *RI*.

Matrix Order	1	2	3	4	5	6	7	8	9
*RI* values	0.00	0.00	0.58	0.90	1.12	1.24	1.32	1.41	1.45

**Table 3:** Classification Criteria of Coupling Coordination Degree.

The Interval of Coupling Co-Ordination Degree *D* Value	Coordination Level	Coupling Coordination Degree
(0.0~0.1)	1	Extreme disorders
(0.1~0.2)	2	Serious disorders
(0.2~0.3)	3	Moderate disorders
(0.3~0.4)	4	Mild disorders
(0.4~0.5)	5	Endangered disorders
(0.5~0.6)	6	Reluctant coordination
(0.6~0.7)	7	Primary coordination
(0.7~0.8)	8	Intermediate coordination
(0.8~0.9)	9	Good coordination
(0.9~1.0)	10	Excellent coordination

**Table 4:** Normalized data of each indicator.

Standard Layer	Index Layer	2017	2018	2019	2020	2021
B_1_	C_1_	0.4064	0.0802	0.0107	0	1
	C_2_	0	0	0.5	0.5	1
	C_3_	0	0.3333	0.3333	1	1
	C_4_	0	0	0.3333	1	1
B_2_	C_5_	0	0.4704	1	0.2069	0.7293
	C_6_	0	0.1170	0.4362	0.9149	1
	C_7_	0	0	0.5	0.5	1
	C_8_	0	0	0.5	1	1
B_3_	C_9_	0	0	0.2	0.8	1
	C_10_	0	0.4484	0.7050	0.8525	1
	C_11_	0.5	0	0.0250	1	0.5300
	C_12_	0	0.625	0.625	1	1
B_4_	C_13_	0	0.3333	0.3333	1	0.3333
	C_14_	0	0	0.5	1	1
	C_15_	0	1	0	0	1
B_5_	C_16_	0	0.2857	0.5714	1	1
	C_17_	0	0	0.6667	1	1
	C_18_	0	0.5	0.5	1	1
	C_19_	0	0	0.5	0.5	1
	C_20_	0	0.0488	0.1870	1	0.0325

**Table 5:** Comprehensive weight calculation results of evaluation indicators.

Target Layer	The Standard Layer	Comprehensive Weight of First-Level Index	Index Layer	Index Layer Weight	Subjective Weight of AHP	Objective Weight of CRITIC	Comprehensive Weight of Secondary-Level Index
A	B_1_	0.2370	C_1_	0.4768	0.1032	0.0853	0.0978
			C_2_	0.1721	0.0516	0.0358	0.0469
			C_3_	0.1738	0.0516	0.0324	0.0458
			C_4_	0.1773	0.0516	0.0347	0.0465
	B_2_	0.2163	C_5_	0.4389	0.0806	0.0886	0.0830
			C_6_	0.2906	0.0806	0.0299	0.0654
			C_7_	0.1463	0.0368	0.0358	0.0365
			C_8_	0.1242	0.0300	0.0349	0.0315
	B_3_	0.2604	C_9_	0.3010	0.1050	0.0324	0.0832
			C_10_	0.2937	0.1050	0.0333	0.0835
			C_11_	0.2968	0.0568	0.0660	0.0595
			C_12_	0.1085	0.0328	0.0376	0.0342
	B_4_	0.1512	C_13_	0.3807	0.0588	0.0531	0.0571
			C_14_	0.1776	0.0374	0.0349	0.0366
			C_15_	0.4417	0.0237	0.1363	0.0575
	B_5_	0.1349	C_16_	0.1642	0.0315	0.0309	0.0313
			C_17_	0.1850	0.0315	0.0347	0.0324
			C_18_	0.2523	0.0105	0.0518	0.0229
			C_19_	0.0749	0.0105	0.0358	0.0181
			C_20_	0.3236	0.0105	0.0760	0.0301

**Table 6:** Calculation Results of TOPSIS Green Mine Construction Assessment in 2017–2021.

Year	Positive Ideal Solution Distance	Distance of Negative Ideal Solution	Relative Proximity
2017	0.932	0.192	0.171
2018	0.820	0.349	0.298
2019	0.671	0.487	0.416
2020	0.488	0.779	0.615
2021	0.270	0.906	0.770

**Table 7:** Analysis of Obstacles of Evaluation Indicators in 2017–2021.

Year	Obstacle Factor 1	Obstacle Factor 2	Obstacle Factor 3	Obstacle Factor 4
2017	C_10_/0.0920	C_9_/0.0917	C_5_/0.0915	C_6_/0.0721
2018	C_1_/0.1148	C_9_/0.1061	C_11_/0.0759	C_6_/0.0734
2019	C_1_/0.1563	C_9_/0.1074	C_11_/0.0932	C_15_/0.0928
2020	C_1_/0.3190	C_5_/0.2138	C_15_/0.1875	C_2_/0.0765
2021	C_13_/0.3246	C_20_/0.2479	C_11_/0.2373	C_5_/0.1901

## Data Availability

Due to the nature of this research, participants of this study did not agree for their data to be shared publicly, so supporting data is not available.
